# An Unsupervised Approach to Predict Functional Relations between Genes Based on Expression Data

**DOI:** 10.1155/2014/154594

**Published:** 2014-03-31

**Authors:** Md. Altaf-Ul-Amin, Tetsuo Katsuragi, Tetsuo Sato, Naoaki Ono, Shigehiko Kanaya

**Affiliations:** Computational Systems Biology Lab, Nara Institute of Science and Technology, Ikoma, Nara 630-0192, Japan

## Abstract

This work presents a novel approach to predict functional relations between genes using gene expression data. Genes may have various types of relations between them, for example, regulatory relations, or they may be concerned with the same protein complex or metabolic/signaling pathways and obviously gene expression data should contain some clues to such relations. The present approach first digitizes the log-ratio type gene expression data of *S. cerevisiae* to a matrix consisting of 1, 0, and −1 indicating highly expressed, no major change, and highly suppressed conditions for genes, respectively. For each gene pair, a probability density mass function table is constructed indicating nine joint probabilities. Then gene pairs were selected based on linear and probabilistic relation between their profiles indicated by the sum of probability density masses in selected points. The selected gene pairs share many Gene Ontology terms. Furthermore a network is constructed by selecting a large number of gene pairs based on FDR analysis and the clustering of the network generates many modules rich with similar function genes. Also, the promoters of the gene sets in many modules are rich with binding sites of known transcription factors indicating the effectiveness of the proposed approach in predicting regulatory relations.

## 1. Introduction

The cell works as a system governed by integrated action of the genes indicating that genes are functionally related; for example, they may have regulatory relations between each other or they may be concerned with the same protein complex or metabolic/signaling pathways and so on. Determining functional relations between genes enables development of a genetic network which leads to the prediction of the complex rolls of the genes in different systems in the cell. Nucleotide and/or amino acid sequence similarities have been extensively used to predict functional relation between genes [1, 2]. Affinity purification [3, 4] and yeast two-hybrid assays [5, 6] are employed to determine physical association between proteins which are gene products. Synthetic lethal screens [7] measure the tendency for genes to compensate the loss of other genes. Scientists have performed numerous studies in an attempt to better understand and classify digenic epistatic relationships [8]. In [9] a probabilistic functional network of yeast genes was constructed by integrating diverse genomic data. In [10] an algorithm was proposed for regulatory networks of gene modules that combines information from genome wide location and expression data sets. Constraint-based Bayesian Structure Learning (BSL) techniques, namely, (a) PC Algorithm, (b) Grow-shrink (GS) algorithm, and (c) Incremental Association Markov Blanket (IAMB), were used to model the functional relationships between genes associated with differentiation potential of aged myogenic progenitors in the form of acyclic networks from the clonal expression profiles [11]. Attempts have been made not only to determine functional relationship between individual genes but also to measure functional relationship between gene sets [12]. Many more similar studies can be cited. Microarray gene expression data incorporating with other information have been extensively used for predicting regulatory relation between genes [13–15]. However it is logical to assume that expression data contains information about various types of functional relations between genes. In the present work we propose an approach for estimating integrated linear and probabilistic relations between expression profiles of genes and applied the concept to determine functional relations between yeast genes solely based on gene expression data. The proposed method successfully detected functionally related gene pairs that share many GO terms. The method also shows promise to be utilized in the process of detecting regulatory relations between genes.

## 2. Materials and Methods

### 2.1. Data Used in This Work

The data used in this work was previously used in other works [16–19]. The data is a 2467 × 79 matrix containing some missing values. Each data point produced by a DNA microarray hybridization experiment represents the log of the ratio of expression levels of a particular gene under two different experimental conditions. The result, from an experiment with *n* genes on a single chip, is a series of *n* log-transformed expression-level ratios. Typically, the numerator of each ratio is the expression level of the gene in the varying condition of interest, whereas the denominator is the expression level of the gene in some reference condition. The expression measurement is positive if the gene is induced (turned up) with respect to the reference state and negative if it is repressed (turned down). The data were collected at various time points during the diauxic shift, the mitotic cell division cycle, sporulation, and temperature and reducing shocks.

### 2.2. Missing Value Imputation

In microarray gene expression data missing values often occur due to various reasons, such as insufficient resolution, image corruption, dust, or scratches on the slide. Usually, microarray datasets are estimated to have more than 5% missing values and up to 90% of genes are affected [20, 21]. The gene expression data considered in this work contains 3760 missing values. The missing values were filled based on principal component analysis (PCA) by using the *R* package pcaMethods [22]. Using PCA we can model a matrix *M* by defining two parameter matrices, the scores, *T*, and the loadings, *P*, such that when multiplied with each other they well reconstruct the original matrix as follows:

(1) $$\begin{array}{c} M=1\times \overset{-}{m}+TP^{t}+E, \end{array}$$

where *E* is the error matrix and $1\times \overset{-}{m}$ denotes the original variable averages. Now if *M* contains missing values but *P* and *T* can be completely estimated, then we can use

(2) $$\begin{array}{c} \hat{M}=1\times \overset{-}{m}+TP^{t} \end{array}$$

as an estimate for *M*_*ij*_ if *M*_*ij*_ is missing.

### 2.3. Digitization of Gene Expression Matrix

After missing value imputation, let us denote the gene expression data matrix as *M*. For each row of *M* we calculate the average and standard deviation. Let for the *i*th row the average and standard deviations be denoted as avg_*i*_ and sd_*i*_. Now, the digitized matrix *D* is created as follows:

(3) $$\begin{array}{c} D_{ij}=1\;\text{if}\;M_{ij}\geq \;\text{av}{\text{g}}_{i}+\text{th}\times {\mathrm{sd}}_{i}\\ D_{ij}=-1\;\text{if}\;M_{ij}\leq {\text{avg}}_{i}-\text{th}\times {\mathrm{sd}}_{i}\\ D_{ij}=0\;\text{otherwise}. \end{array}$$

**Figure 1:**
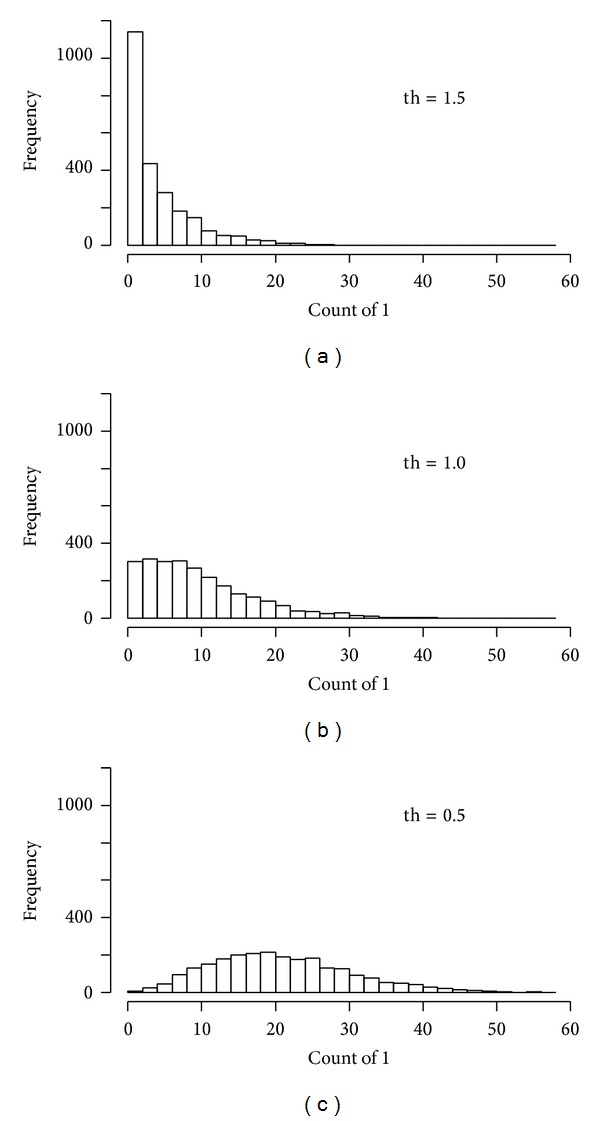
Distribution of the genes with respect to the count of 1 in their profiles in the context of the digitized matrix.

In the above equations “th” is a threshold which should be a real number and in most practical cases it is within 0 to 2. We digitized the data using the values of threshold “th” as 0.5, 1, and 1.5. For each case the distribution of the genes with respect to the count of 1 s in their profiles is shown in Figure 1. In case of th = 0.5, the distribution approaches roughly normal and we observed similar trend in case of −1. Hence in this work we considered th = 0.5 for the digitization of the gene expression data.

### 2.4. Probability Density Mass Function Table

Based on a digitized matrix containing only 1, 0, and −1 a probability density mass function table can be constructed corresponding to each gene pair indicating nine joint probabilities as shown in Table 1.

Any element of the above table *P*(*k*, *k*′) (corresponding to two genes say, gene *a* and gene *b*) where *k*, *k*′ ∈ {1, 0, − 1} can be calculated by assuming TRUE = 1 and FALSE = 0 in (4) as follows:

(4) $$\begin{array}{c} P\left(k,k^{\prime }\right)=\frac{\sum_{i=1}^{N}D_{ai}==k\;\text{AND}\;D_{bi}==k^{\prime }}{N}. \end{array}$$

Here *N* is the width of matrix *D*.

We assume that the joint probabilities of Table 1 and corresponding conditional probabilities contain important clues to estimate functional relations between genes.

### 2.5. Hypothesis

In this work we hypothesize that when gene *a* is positively functionally related to gene *b*, then *P*(*b* = 1 | *a* = 1) should be statistically high. Using Bayes rule we can write *P*(*b* = 1 | *a* = 1) = *P*(*a* = 1, *b* = 1)/*P*(*a* = 1). Now if *P*(*a* = 1) is very small, then *P*(*b* = 1 | *a* = 1) can be very high and that can sometimes happen because of noisy data. To avoid this problem we can consider *P*(*b* = 1, *a* = 1) as an indicator that gene *a* is positively functionally related to gene *b*. To further strengthen the case we consider that when both *P*(*b* = 1, *a* = 1) and *P*(*b* = 1, *a* = 1) + *P*(*b* = 0, *a* = 0) + *P*(*b* = −1, *a* = −1) are statistically significant then gene a and gene b are positively functionally related. Considering other joint probability masses might be useful for finding functional relations between some multi function genes. By intuition we can realize that the sum of probabilities *P*(*b* = 1, *a* = 1) + *P*(*b* = 0, *a* = 0) + *P*(*b* = −1, *a* = −1) actually indicates an integrated measure of both linear and probabilistic relations between the profiles of two genes and this term will be referred to as positive linear and probabilistic relation (LPR_pos_) in the following. To our knowledge this is the first approach to measure similarity between two multivariate entities based on joint probability density masses in selected points giving emphasis on both linear and probabilistic relations.

## 3. Results

### 3.1. Effectiveness of LPRpos

**Figure 2:**
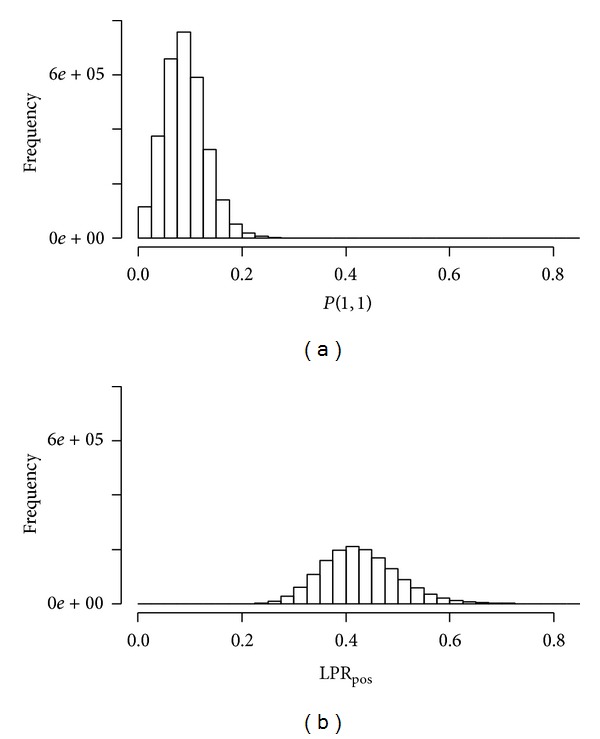
Distribution of gene pairs in the context of (a) *P*(1,1) and (b) LPR_pos_.

The distribution of all gene pairs in the context of *P*(1,1) is shown in Figure 2(a). The average value of *P*(1,1) is 0.0819. We calculated LPR_pos_ for the gene pairs for which *P*(1,1) is larger than the average value. The distribution of those gene pairs with respect to LPR_pos_ is shown in Figure 2(b). The average value of LPR_pos_ is 0.429. Initially we selected the highest 1%, 2%, 3%, 4%, and 5% gene pairs from the distribution of Figure 2(b), that is, gene pairs with higher LPR_pos_ values, and determined the number of GO terms [23] shared by both the genes of each pair.

**Figure 3:**
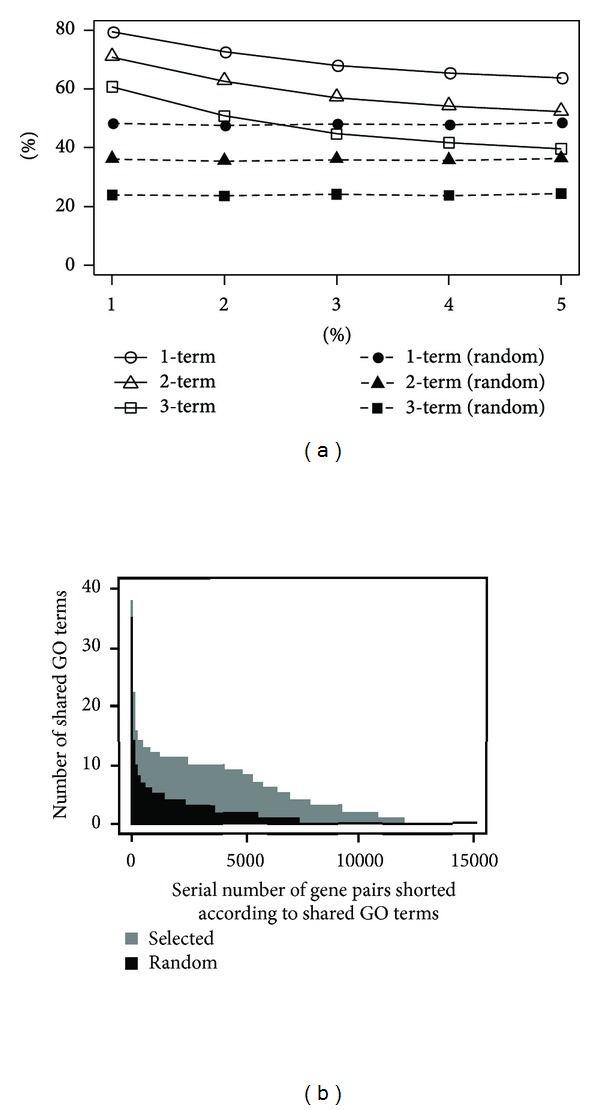
(a) *x*-axis is percentage of gene pairs of the distribution of Figure 2(b) selected based on higher LPR_pos_ values and *y*-axis is percentage of selected gene pairs that share at least 1, 2, or 3 GO terms. Empty markers correspond to gene pairs selected by the proposed method and filled markers corresponding to equal number of randomly selected gene pairs. (b) Actual number of GO terms shared by selected and random gene pairs corresponding to the 1% point of (a).

Figure 3(a) shows the percentage of selected gene pairs that share at least 1, 2, and, 3 GO terms and also that of equal number of randomly selected gene pairs. In the context of minimum number of shared GO terms the percentage of selected gene pairs is always much higher compared to that of randomly selected pairs. Figure 3(a) further shows that the higher the lower cutoff value of LPR_pos_ for a group of gene pairs is, the higher proportion of the gene pairs share common GO terms. To further illustrate the result we show in Figure 3(b) the actual number of shared GO terms for the highest 1% selected gene pairs and the equal number of random gene pairs which implies that the gene pairs selected based on LPR_pos_ share much more GO terms. Thus LPR_pos_ is a good measure to determine functional relation between genes.

### 3.2. FDR Analysis

We conducted FDR (false discovery rate) [24, 25] analysis to statistically assess the false positive rates among the selected gene pairs based on LPR_pos_. For each pair of genes for which *P*(1, 1) is above average we did the following.

(i) The numbers of 1 s, 0 s, and −1 s in the digital profile of both genes are counted.
(ii) Random profiles of both the genes are constructed by randomly imputing the same numbers of 1 s, 0 s, and −1 s. This process is repeated 100 times.
(iii) Then, *C*(1,1), *C*(0,0), and *C*(−1, −1) are calculated for both real and random profile pairs. *C*(*k*, *k*){*k* ∈ 1, 0, − 1} is the total number of profile points for which the expression level of both genes is *k*. In case of random profiles the average values corresponding 100 random profile pairs were considered.
(iv) A chi-square value is calculated as follows where *N* is the width of the expression matrix:
  (5) χ2=[∑k=1,0,−1{C(k,k)real−C(k,k)random}2C(k,k)random] +{∑k=1,0,−1C(k,k)real−∑k=1,0,−1C(k,k)random}2N−∑k=1,0,−1C(k,k)random.
(v) Based on the chi-square value, a *P*-value for the gene pair is determined using *R* statistical software. Note that LPR_pos_ is directly proportional to ∑_*k*=1,0,−1_ *C*(*k*, *k*)_real_.

**Figure 4:**
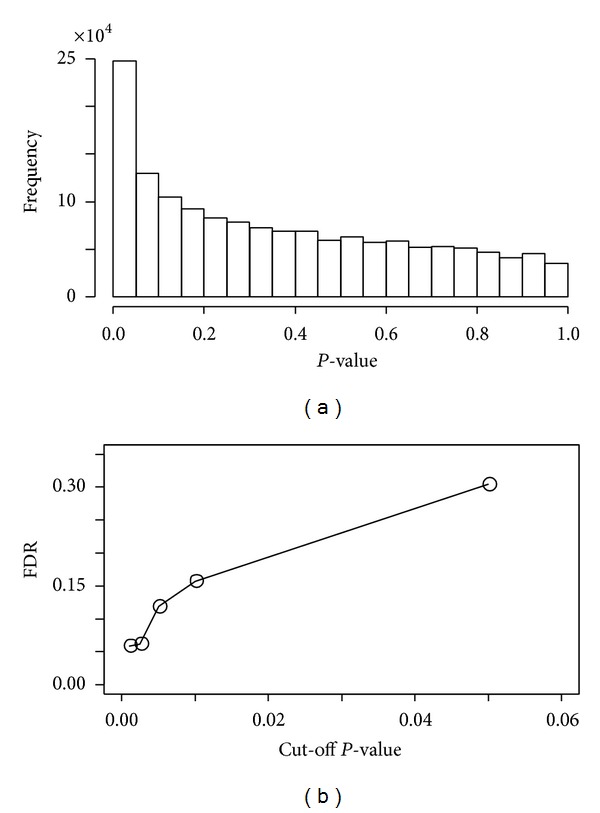
(a) Distribution of the gene pairs with respect to the *χ*-square *P*-values. (b) Plot of FDR with respect to cutoff *P*-values.

Figure 4(a) shows the distribution of the gene pairs with respect to the *P*-values with a *P*-value interval of 0.05. For any given cutoff *P*-value the FDR is calculated as follows:

(6) FDR=(Total  #  of  gene  pairs)×(P-value)cut-off#  of  gene  pairs  with  P-value  less  than  (P-value)cut-off.

Figure 4(b) shows the plot of FDR with respect to cutoff *P*-values. As the cutoff *P*-value decreases, FDR decreases rapidly and becomes roughly constant at *P*-value of 0.001. There are 25559 gene pairs for which the *P*-value is less than 0.001.

### 3.3. Network and Modules of the Selected Gene Pairs

Based on the FDR analysis of the above section, we selected 25559 gene pairs having highest LPR_pos_ values. Such selected gene pairs make a network consisting of 2131 nodes. We determined high density modules in that network using the network clustering algorithm DPClusO [26] and found 1154 modules of size 3 or more (see Supplementary File  1 in supplementary material available online at http://dx.doi.org/10.1155/2014/154594).

#### 3.3.1. Richness of Similar Function Genes

**Figure 5:**
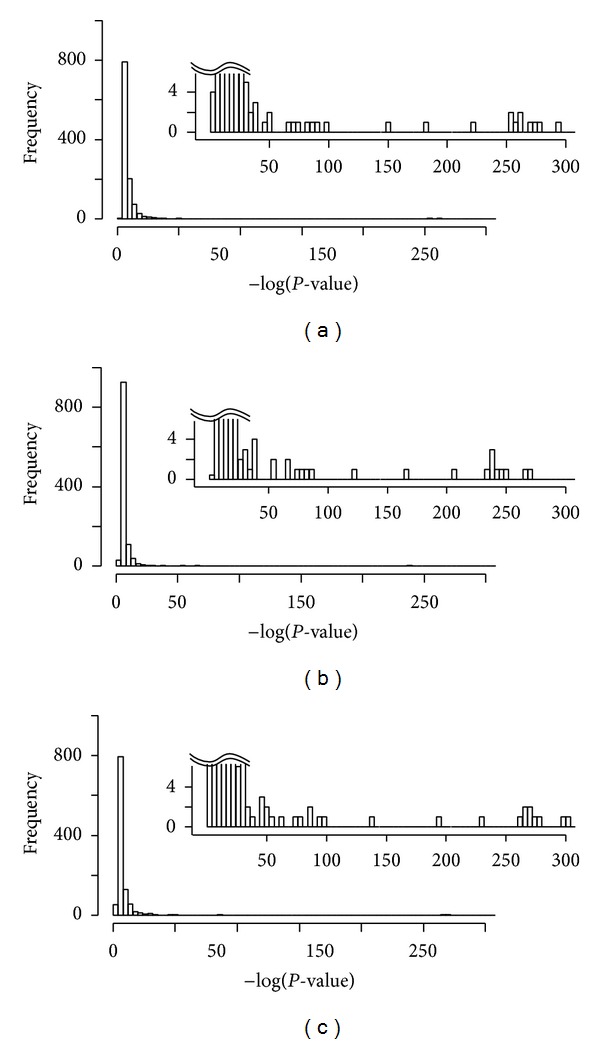
Distribution of the modules with respect to −log(*P*-value). *P*-values determined in the context of all three types of GO terms (a) biological process (BP), (b) molecular function (MF), and (c) cellular compartment (CC). The lower part of each graph is enlarged in the insets.

To evaluate the richness of similar function genes in the modules we calculated their hypergeometric *P*-values by using the *R* package GOstats [27] in the context of all three types of GO terms: biological process (BP), cellular compartment (CC), and molecular function (MF). Figures 5(a), 5(b), and 5(c) show the distribution of the modules with respect to −log(*P*-value) which implies that almost all the modules are statistically significant. We selected 10 lowest *P*-value clusters corresponding to different GO terms from each of the three distributions of Figure 5 and their set union resulted in 22 clusters. Some biological information from the SGD database [28] about those 22 clusters is summarized in Table 2. Column 3 in Table 2 shows the *P*-values and corresponding GO terms determined by GOstats. Column 4 in Table 2 shows other GO terms retrieved from SGD database associated to the clusters covering many genes which implies that almost all the genes of each of the clusters could be associated to important GO terms which confirms the fact that the proposed method is a promising way to establish functional relation between genes based on expression data.

#### 3.3.2. Richness of Similar Binding Sites

Furthermore to verify the presence of similar binding sites in the promoters of the genes included in individual modules we used the tool PRIMA (PRomoter Integration in Microarray Analysis) [29] from the software package EXPANDER [30]. Total 180 modules were found to have *P*-values less than 10^−3^ in the context of binding site enrichment of 57 various transcription factors. The enrichment table generated by EXPANDER is in supplementary material (Supplementary Table  1).  Table 3 shows information about 10 modules corresponding to lowest *P*-values involving 10 different transcription factors. We downloaded a list of known regulatory relations from the YEASTRACT database [31] and verified whether the genes in a module have regulatory relation with the associated transcription factor. Column 6 of Table 3 shows that a large number of genes in individual modules are already reported to be regulated by the corresponding transcription factor. Only in case of CID736, though all 3 genes contain in their promoters the binding site of the transcription factor DAL82, no regulatory relation between those genes is reported in the YEASTRACT database presently. However based on our analysis regulatory relations between DAL82 and those three genes may be predicted. Thus the proposed measure can also be integrated to other types of information for developing a method to predict regulatory relations between genes which is one of our future works.

## 4. Conclusions

In this work we propose a novel measure to determine functional relation between genes based on gene expression data. The present approach first digitizes the log-ratio type gene expression data to a matrix consisting of 1, 0, and −1 indicating highly expressed, no major change and highly suppressed conditions for genes, respectively. Then a probability density mass function table is constructed indicating nine joint probabilities for each pair of genes. Those pairs of genes were considered as functionally related for which the sum of probability density masses in selected points are statistically significant. We applied the method to a sample gene expression data of* S. cerevisiae. *It was found that substantial majority of the selected gene pairs share many GO terms. Also the network consisting of the selected gene pairs contains high density modules. It was shown that those modules were rich with similar function genes. Furthermore, it was verified that for many modules many of the genes contain similar binding sites in their promoters corresponding to known transcription factors of yeast and those transcription factors are known regulators of many of the genes in the corresponding module. Above all this work introduces a new approach for simultaneously measuring both linear and probabilistic relations between multivariate entities which is useful for handling multivariate data and big data biology.

## Supplementary Material

Supplementary Table 1: The tool PRIMA (PRomoter Integration in Microarray Analysis) from the software package EXPANDER was used to verify the presence of similar binding sites in the promoters of the genes included in individual modules. Total 180 modules were found to have p-values less than 10-3 in the context of binding site enrichment of 57 various transcription factors. One module may be associated to more than one transcription factor. Supplementary Table 1 is the enrichment table generated by Expander.

## Figures and Tables

**Table 1:** Nine joint probabilities calculated for each gene pair.

*a*/*b*	1	0	−1
1	*P*(1, 1)	*P*(1, 0)	*P*(1, −1)
0	*P*(0, 1)	*P*(0, 0)	*P*(0, −1)
−1	*P*(−1, 1)	*P*(−1, 0)	*P*(−1, −1)

**Table 2:** Richness of similar function genes in selected clusters. For each cluster, hypergeometric *P*-values, corresponding GO terms, and also the actual number of genes of a particular function are indicated.

CID	Total number of genes	*P*-value/GO ID (From GOstats result)	Some relevant GO terms (corresponding number of genes) (From SGD database)
4	97	1.20*E* − 131/GO:0022626 (CC) 2.62*E* − 117/GO:0002181 (BP) 4.80*E* − 129/GO:0003735 (MF)	Cytosolic ribosome (94), structural constituent of ribosome (94), cytoplasmic translation (93), ribosome (96)

16	76	6.42*E* − 24/GO:0044391 (CC) 7.23*E* − 17/GO:0006412 (BP)	Ribosomal subunit (37), structural molecule activity (38)

19	73	3.29*E* − 23/GO:0030529 (CC)	Ribonucleoprotein complex (47), intracellular part (73)

226	8	1.50*E* − 20/GO:0000788 (CC) 1.93*E* − 14/GO:0006333 (BP)	Nuclear nucleosome (8), DNA bending complex (8)

1	113	1.42*E* − 17/GO:0042254 (BP)	Cellular metabolic process (104), intracellular part (109)

44	34	2.89*E* − 16/GO:0005840 (CC)	Cytosolic part (21), cytoplasm (34)

35	44	3.35*E* − 16/GO:0010467 (BP)	Gene expression (41), primary metabolic process (43)

85	17	4.76*E* − 14/GO:0044429 (CC)	Mitochondrial part (14), mitochondrion (16)

155	11	6.28*E* − 14/GO:0051082 (MF) 4.97*E* − 13/GO:0006457 (BP)	Protein folding (9), protein binding (11), cellular protein metabolic process (10)

278	7	3.00*E* − 13/GO:0000502 (CC)	Proteasome complex (7), proteasome storage granule (5)

87	16	5.26*E* − 13/GO:0005730 (CC)	Nucleolus (12), non-membrane-bounded organelle (14)

107	14	1.97*E* − 12/GO:0007005 (BP)	Mitochondrion organization (12), cellular component organization (13)

121	13	5.32*E* − 12/GO:0006094 (BP)	Glycolysis (7), generation of precursor metabolites and energy (9)

442	5	1.55*E* − 11/GO:0022904 (BP)	Mitochondrial respiratory chain (5), oxidoreductase complex (5)

173	10	1.56*E* − 11/GO:0006457 (BP) 2.58*E* − 08/GO:0051082 (MF)	Protein folding (7), unfolded protein binding (5), protein binding (8)

282	7	5.58*E* − 11/GO:0004298 (MF)	Modification-dependent protein catabolic process (7), roteasomal ubiquitin-independent protein catabolic process (5)

71	15	5.90*E* − 11/GO:0005840 (CC)	Ribosome (13), ribonucleoprotein complex (14)

725	3	1.61*E* − 09/GO:0003993 (MF)	Acid phosphatase activity (2)

214	9	2.88*E* − 09/GO:0008121 (MF)	Hydrogen ion transmembrane transporter activity (5), single-organism metabolic process (7)

736	3	4.03*E* − 09/GO:0004067 (MF)	Asparaginase activity (3)

1092	3	2.26*E* − 08/GO:0015002 (MF)	Heme-copper terminal oxidase activity (3)

270	7	2.32*E* − 08/GO:0015078 (MF)	Ion transmembrane transporter activity (6)

**Table 3:** Richness of binding sites in the promoters of the module genes corresponding to 10 different transcription factors.

CID	Size	TF	Number of Promo. (PRIMA)	*P*-value	Known regulatory relations (YEASTRACT)
3	98	YP00066 [SFP1]	58	2.82*E* − 42	98
5	95	M00213 [RAP1]	55	3.82*E* − 28	93
72	18	YP00036 [MBP1]	10	4.40*E* − 12	12
155	11	M00169 [HSF]	7	2.38*E* − 09	11
230	8	YP00068 [SIP4]	5	7.89*E* − 09	4
227	8	YP00064 [RPN4]	8	1.01*E* − 08	8
725	3	M00064 [PHO4]	3	1.08*E* − 08	3
259	7	YP00076 [STB1]	5	8.97*E* − 08	2
736	3	YP00013 [DAL82]	3	3.65*E* − 07	0
233	8	YP00043 [MSN4]	8	1.03*E* − 06	7
