# Supplementary material for: An Unsupervised Approach to Predict Functional Relations between Genes Based on Expression Data
Source: Biomed Res Int. 2014 Mar 31;2014:154594. doi: 10.1155/2014/154594 (PMC3988973; doi:10.1155/2014/154594)
Supplement: Supplementary file 1 — Supplementary Table 1: The tool PRIMA (PRomoter Integration in Microarray Analysis) from the software package EXPANDER was used to verify the presence of similar binding sites in the promoters of the genes included in individual modules. Total 180 modules were found to have p-values less than 10-3 in the context of binding site enrichment of 57 various transcription factors. One module may be associated to more than one transcription factor. Supplementary Table 1 is the enrichment table generated by Expander. [file 154594.f1.docx]

Supplementary File 1: Based on the FDR analysis, 25559 gene pairs having highest LPRpos values were selected. Such selected gene pairs make a network consisting of 2131 nodes. Total 1154 high density modules of size 3 or more were determined based on that network using the clustering algorithm DPClusO. Supplementary File 1 contains 1154 lines and each line contains the list of genes belonging to a module.

Supplementary Table 1: The tool PRIMA (PRomoter Integration in Microarray Analysis) from the software package EXPANDER was used to verify the presence of similar binding sites in the promoters of the genes included in individual modules. Total 180 modules were found to have p-values less than 10-3 in the context of binding site enrichment of 57 various transcription factors. One module may be associated to more than one transcription factor. Supplementary Table 1 is the enrichment table generated by Expander.
